# The Wise Teacher

**Published:** 1870-01

**Authors:** 


### THE WISE TEACHER.

Our wealthiest and best New Yorkers generally understand that there is no better private school in the United States for lads than Professor Charlier's, who is, perhaps, the most successfult eacher of youth in the United States; successful, because he makes scholars and gentlemen of them, and in addition, seeks to impress upon their minds in the most indelible manner, not only the importance of caring for their health, but how to do it. For some years past he has been in the habit of making selections from our Health Tracts, and presenting a series to each scholar, not merely requiring them to be read, but to be recopied, and if they are learning another language—French or Latin—he requires them to translate these tracts, and thus compels them to study them to get their full meaning with the result of their never being forgotten in all after life. For want of such inculcations many a noble youth, the pride of his home, has entered his professional career only to dwindle away and die within a very few months, and that, too, from the want of a very trifling amount of knowledge in reference to the preservation of health. The selections of the Professor for 1869 were: 1. Attention to the feet; 2. Regulating the Bowels; 3. How to eat wisely; 4.

How to cure a cold; 5. Health's three essentials; 6. Taking care of the teeth; 7. Sleeping; 8. Danger of cooling off too quickly, or checking perspiration. Sent by mail for ten cents.

